# Are High-Impact Species Predictable? An Analysis of Naturalised Grasses in Northern Australia

**DOI:** 10.1371/journal.pone.0068678

**Published:** 2013-07-09

**Authors:** Rieks D. van Klinken, F. Dane Panetta, Shaun R. Coutts

**Affiliations:** 1 CSIRO Ecosystem Sciences, CSIRO, Brisbane, Queensland, Australia; 2 Melbourne School of Land and Environment, The University of Melbourne, Melbourne, Victoria, Australia; 3 NERP Environmental Decisions Hub, Centre for Biodiversity and Conservation Science, University of Queensland, Brisbane, Queensland, Australia; University of Konstanz, Germany

## Abstract

Predicting which species are likely to cause serious impacts in the future is crucial for targeting management efforts, but the characteristics of such species remain largely unconfirmed. We use data and expert opinion on tropical and subtropical grasses naturalised in Australia since European settlement to identify naturalised and high-impact species and subsequently to test whether high-impact species are predictable. High-impact species for the three main affected sectors (environment, pastoral and agriculture) were determined by assessing evidence against pre-defined criteria. Twenty-one of the 155 naturalised species (14%) were classified as high-impact, including four that affected more than one sector. High-impact species were more likely to have faster spread rates (regions invaded per decade) and to be semi-aquatic. Spread rate was best explained by whether species had been actively spread (as pasture), and time since naturalisation, but may not be explanatory as it was tightly correlated with range size and incidence rate. Giving more weight to minimising the chance of overlooking high-impact species, a priority for biosecurity, meant a wider range of predictors was required to identify high-impact species, and the predictive power of the models was reduced. By-sector analysis of predictors of high impact species was limited by their relative rarity, but showed sector differences, including to the universal predictors (spread rate and habitat) and life history. Furthermore, species causing high impact to agriculture have changed in the past 10 years with changes in farming practice, highlighting the importance of context in determining impact. A rationale for invasion ecology is to improve the prediction and response to future threats. Although our study identifies some universal predictors, it suggests improved prediction will require a far greater emphasis on impact rather than invasiveness, and will need to account for the individual circumstances of affected sectors and the relative rarity of high-impact species.

## Introduction

Many invasive plants cause substantial environmental, economic and social impacts [1], [2], [3]. Invasive plants represent the subset of imported species that successfully naturalise and spread [4]. Considerable effort has been devoted to explaining and predicting, on the basis of plant traits, origin and propagule pressure, which species are likely to be most invasive [5], [6]. However, invasiveness as an ecological phenomenon, and impact defined as the ecological, social, and economic consequences of invaders, although frequently confounded [4], [7], [8], are distinct concepts [4]. In fact limited research suggests that invasiveness (measured as mean rate of spread) is a poor predictor of impact across diverse taxa [7]. Predicting which species will ultimately become problematic, as opposed to being invasive *per se*, remains difficult and is largely overlooked [9], [10], [11]. New invasions continue, so it is particularly critical to anticipate which species will cause greatest impact. In this paper we test whether there are predictors for species among tropical and subtropical grasses that have naturalised in Australia that went on to cause serious impact.

The term ‘weed’ is suggestive of impact but has often been used synonymously with ‘naturalised species’ in the literature, and consequently is not useful in categorising impact. For example, we found that of the 155 naturalised tropical and subtropical grasses in Australia, 98.7% have been reported as a weed overseas and 93.5% in Australia (Table S1). Furthermore, most definitions of impact have focussed on ecological effects of plant invasions, such as nutrient cycling and hydrology [9], [12], rather than impacts that specifically affect environmental, economic or social values that might be the target for management and policy responses [13]. For example, ‘transformers’ have been defined without special reference to possible management objectives as “invasive plants that change the character, condition, form or nature of ecosystems over substantial areas” [4]. We therefore developed an evidence-based approach, using predefined criteria, to identify the subset of high-impact species already causing serious impact to the environment, pastoral industry or agricultural industries. Our approach thereby acknowledges that criteria for impact differ with sectors and need to be defined for each. This methodology contrasts with other approaches, such as meta-analysis [12] or data-mining [9] used to describe ecological impacts and their patterns in published quantitative studies. However, it has the advantage of allowing explicit consideration of the context under which invasions are occurring and the types of impact of greatest management concern. Also, published quantitative information on impact is unavailable for most species.

Very few studies have tested species-level predictors of impact, and the issue of whether traits relate to invasiveness or impact *per se* has rarely, if at all, been addressed [9]. A common assumption is that high-impact species are more invasive. High-impact species were faster invaders in China when impact was determined by number of publications [14], [15], but was not significant in a global study that categorised impact according to ecological effects on species populations [7]. Other factors are also expected to be important predictors of impact in particular sectors, although we are unaware of any systematic analyses. For example, high-biomass, often perennial, grasses are known to cause serious environmental impacts through altering the grass-fire cycle [16], many serious pastoral weeds have low palatability or high toxicity [17], and some of the most serious weeds in agricultural systems are the result of the development of herbicide resistance [18].

Exotic grasses in northern Australia offer a good model system for testing predictors of impact because they can cause profound negative impacts to the environment and agriculture [19], [20], [21], [22], [23] and their impacts in northern Australia are particularly severe [20], [23], [24], [25], [26]. Exotic grasses are also diverse in northern Australia, and their importation, naturalisation and impacts there are relatively well documented. This includes maintenance of a Commonwealth Plant Introduction (CPI) list from 1929 to 1997 which records approximately 145,000 plant accessions imported by CSIRO and agricultural agencies and agricultural faculties during that period [27].

We tested whether it was possible to predict which naturalised species became high-impact overall, and by impacted sector (environmental, pastoral and agricultural). Weed risk assessments are typically aimed at preventing introduction of any high-impact species [28], so it makes sense to determine whether there are generic predictors as well as sector-specific ones. We also tested whether any generic predictors of high-impact species were the same as predictors of spread rate. Predictors of impact were included for which data were available for the full set of naturalised species and which we considered might have a bearing on impact and spread rate: namely life history traits, introduction pathway, naturalisation history and spread rate (for impact). When the costs of escaped exotic species vastly outweigh the benefits those species might bring, correctly identifying high-impact species is more important than avoiding labelling a harmless species as high impact [29]. Previous studies have shown that model outcomes can be sensitive to how false positives and false negatives are weighted [30]. We therefore also test whether changing this assumption will affect predictors of high-impact species.

## Methods

A list of tropical and subtropical grass species that had established naturally self-sustaining populations (naturalised) in Australia was compiled using records in the Australian Virtual Herbarium (which includes all Australian herbaria), the literature [21], [31], [32], [33], authoritative web databases and taxonomic expertise (B.K. Simons, Queensland Herbarium). Higher classifications (sub-families and tribes) were based on Kellogg ([34], [35]) and Simon ([36]) and species designations followed Simon & Alfonso ([33]). Grasses were categorised as tropical/subtropical on the basis of their biology and native range distribution (van Klinken *et al*., in prep.). For each species we recorded plant traits, first date of introduction and naturalisation, likely introduction pathway, range and spread rates, whether the species was actively spread and promoted in Australia as pasture or turf, and whether it caused high impact on one or more sectors (Table 1).

**Table 1:** Predictors tested or excluded from model-fitting analyses.

Predictor	Type			Units or levels	Explanation
**Included in analyses**					
*Historical*					
*No. reg	Continuous			No. regions	Number of regions in Australia in which species has been recorded
spr.rate	Continuous			regions/decade	Number of regions in which the species is recorded as naturalized divided by the number of decades since the species first became naturalized.
nat	Continuous			Year	Year the species was first recorded as naturalised in Australia
Act.spr (active spread)	Binary			[no, yes]	Was the species actively spread and promoted by people?
intro	Categorical			5 pathways	The introduction pathway into Australia.
*Biological*					
semi.aqua	Categorical			[no, yes]	Is the species semi-aquatic?
ann.per	Categorical			[annual, perennial, both]	Is the species an annual or a perennial?
tuft	Categorical			[no, yes, variable]	Is the species tufted or not?
rhizo	Categorical			[no, yes, variable]	Does the species have Rhizomes?
stolon	Categorical			[no, yes, variable]	Does the species have stolons?
**Excluded from analyses**					
Native origin	Categorical			7 regions	Native to which of 7 global biogeographic regions
Incidence	Continuous			No. records	Number of herbarium records in Australia
Incidence rate	Continuous			Records/decade	Average number of herbarium records in Australia per decade since naturalised
Photosynthesis pathway	Categorical			[C3,C4]	Photosynthesis pathway

See text for details of the analysis. Genus was always used as a random effect. Predictors only included in the spread rate analysis are indicated by asterisks.

We focused on plant traits that were available for all species and which we considered might have a bearing on spread rate and impact. For each species we recorded life history (annual, perennial, or annual/biennial/perennial), growth habit (tufted, stoloniferous and/or rhizomatous) and habitat preference (terrestrial species or semi-aquatic, thriving in seasonally inundated or waterlogged habitats). Native origin was excluded, as a separate analysis of the same species found no difference in native range between all naturalised species and the high-impact species (van Klinken *et al*., in prep.). Photosynthetic pathway (C_3_ or C_4_) was also excluded from the analysis because there were too few C_3_ grasses (seven species) in the data set to make it a reliable predictor.

A range of sources, including herbarium records, the literature and CPI records, were used to determine the first recorded date, the first recorded date in CPI records, and most likely pathway of introduction into Australia. The most likely introduction pathway was categorised as: pasture or turf, contaminant of imported seeds, crop, ornamental, or unexplained. For some species there were multiple introduction and naturalisation events, and potentially more than one pathway for introduction, in which case the primary pathway was identified based on eventual use. Herbarium records and the literature were consulted to determine when each species was first recorded as naturalised. The naturalised species that were subsequently widely promoted and actively spread in Australia as pasture or turf were identified using the literature [37], [38], [39] and consultation with relevant pasture scientists.

Herbarium records (records collected through to 31 December 2009) were used as the best available estimate of distribution within Australia and to calculate incidence rate (number of records [incidence] per decade since naturalisation). Distribution within Australia was described as the number of Interim Biogeographic Regionalisation of Australia (IBRA Version 4.0) regions, although temperate Tasmania was included as a single biogeographic region (rather than as 10 small, temperate regions), giving a total of 71 regions. Spread rate (number of IBRA regions per decade) was based on the 2009 distribution of each species. Duplicate collections and records that clearly did not represent naturalisation (e.g. those from research stations, glasshouses, botanic gardens, agricultural colleges, demonstration farms and experimental plots) were excluded from the analysis, unless the collection label unambiguously indicated that the species had self-propagated.

### Impact

Evidence for species having high impact on the environment, pastoral industry and agriculture (cropping and horticulture) was assessed against criteria [40] as follows:

#### “Environmental”

Species that have become dominant (defined as percent herbaceous cover) in environmental reserves as a result of natural spread (implying an ability to invade), and not dependent on human related disturbance (e.g. excludes roadsides that are regularly slashed, high-use areas such as campgrounds, and land that has historically had heavy, prolonged grazing). Environmental impact has not been quantified for most grass species, so it was assumed that dominance under these circumstances equated to serious impact [20]. Specific examples meeting these criteria were required for a species to be considered as high-impact.

#### “Pastoral” and “Agricultural”

Species that the respective sector considers as currently having a serious negative impact, and therefore requiring specifically targeted control work, or significantly altered on-farm practice (e.g. change in stock management). We excluded species whose impacts are largely preventable through industry-standard, on-farm practice, and “systems weeds” such as cropping weeds that are managed as part of a suite of competitors.

For each species specific examples of impact which met all the criteria were sought from literature, authoritative websites and unpublished sector reports, and phone-interviews with over 20 targeted professionals (Table S4). Examples were then cross-validated, including by interviewing experts with broad knowledge within a sector and direct knowledge of the reported impacts and the context in which it occurred. The compiled list was then circulated on the “enviroweeds” list-server to identify any omissions which were then followed up further.

### Analysis

Our goal was to find the best set of predictors for which naturalised species became high-impact. Spread rate (regions invaded per decade) was identified as an important predictor (see results), so an additional analysis was undertaken to determine whether the same factors predicted spread rate as impact.

Testing predictors of impact within sector, while still controlling for genus, was constrained by the relatively small number of high impact species. We therefore present quantitative trends for each sector, and results from an analysis for the two sectors (environment and pastoral) for which by-sector analysis was possible.

#### Predictors of high-impact species

We used generalized linear mixed effect models with a binomial error structure to predict the binary variable ‘high-impact’, which was 1 if the species met the criteria in the impact section, and 0 otherwise. The structure of the random effect was very simple, only the intercept for each genus was allowed to vary. This allowed species to be more or less likely to be high-impact based on their genus. We also tested genus nested within tribe, but tribe did not explain any of the variance above that explained by genus, so was dropped from the analysis. Henceforth we refer to these models as glme. Because there were few high-impact species, only nine predictors were used (see Table 1) and no interactions were tested. Date of first naturalisation was used rather than time of introduction as it was considered more likely to be explanatory. Number of regions, incidence and incidence rate were excluded as they were highly correlated with each other and with spread rate (see results). All models were fitted using the ‘lme4’ library ([41], lme4: Linear mixed-effects models using S4 classes) in the statistical computing language R [42].

Model fitting was done in two ways. First we used a standard approach, fitting a separate glme to every unique combination of the nine predictors (n = 512) using the ‘combinations’ function in the ‘gtools’ library ([43], gtools: Various R programming tools). We kept genus as the random effect in all cases. We then compared the performance of each glme using AICc and relative AICc weights, which compare the AICc support for each model [44]. We calculated AICc using the AIC.mer function in the AICcmodavg (Mazerolle, 2013, AICcmodavg: Model selection and multimodel inference based on (Q)AIC(c). R package version 1.30.). This analysis was conducted with the full set of species, and just those species that naturalised on or prior to 1988, the last year of naturalisation for a high-impact species. In a second analysis we used an approach inspired by statistical learning. Instead of using AICc to measure performance we directly tested how good a classifier each glme was using leave-one-out cross validation to estimate misclassification rates. Each row in the dataset represented one species and consisted of the set of predictors in Table 1, the genus of the species, and if it was high impact. One at a time, 134 rows (out of 155 rows) in the dataset were held out (explanation of which rows follows) and a glme was fit to the remaining 154 row dataset. That glme was applied to the held-out species and used to predict the probability that it was a high impact species. We could not use all 155 species as hold-out species because 21 were the only representative of their genus in the data set. This meant that if they were held out the glme would be fitted without that genus, and thus prediction on the held-out species would be impossible.

We measured how well each glme worked as a classifier using *Weuc*, the weighted Euclidean distance between the glme and a hypothetical ‘perfect classifier’ [45].
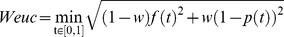
(1)where *t* is the classification threshold, a number between 0 and 1, above which a probability is classified true. *f*(*t*) and *p*(*t*) are the false positive and true positive rates at a given threshold, *t*.



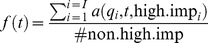
(2a)

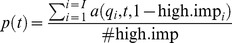
(2b)

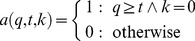
(2c)


Where *q_i_* is the probability of species *i* being a high-impact species and is estimated using the cross validation outlined above, *I* is the total number of species for which a probability could be estimated (*I* = 134). high.imp*_i_* is 1 if species *i* is high-impact and 0 if not. *a*(*q, t, k*) is a function that is either: 1 if the species is falsely predicted to be a high-impact species given classification threshold *t* and 0 otherwise (*k* = high.imp*_i_*); or 1 if species *i* is correctly classified as a high-impact species given classification threshold *t*, and 0 otherwise (*k* = 1–high.imp*_i_*). #non-high.imp is the total number of non-high impact species among species for which a prediction could be made. Finally, #high.imp is the total number of high impact species among species for which a prediction could be made. *w* is the relative weight given to true positives versus false positives; if *w = *1 we do not care about false positives and only try to maximise true positives, if *w* = 0 we only try to minimise false negatives, and when *w* = 0.5 we give the two types of errors equal weight.

In the context of invasive species, those species which do become highly damaging are generally difficult to control and costly to a large number of people, thus, we may tolerate a high false positive rate to achieve a high true positive rate. AIC implicitly assumes equal weighting of true and false positives. We scaled *Weuc* so that it lies between 0 (perfect classifier) and 1 (random guessing) for all values of *w*. We tested two values of *w*, *w* = 0.5 (equal weight) and *w = *0.9 (true positives weighted more heavily).

To explore the effect of important predictors we used a bootstrap procedure to estimate uncertainty around the coefficients of the best supported model, logit(Pr[high.impact])∼spr.rate+semi.aqua+(1|genus). For each genus we randomly selected the same number of rows from the data set with replacement as there were species in that genus. This ensured that the number of species within each genus remained the same between resamples. Resampled data that contained fewer than 15 high-impact species were rejected and redrawn, to ensure the glme fitting would converge. A glme was then fitted to the resampled data set, the intercept and the coefficients for spr.rate and semi.aqua were recorded for each genus. This process was repeated 10,000 times to generate distributions of intercepts and coefficients, from which means and 95 percent confidence intervals were taken.

#### Predictors of spread rate

To determine which factors influenced spread rate we used glmes to predict log(spr.rate) for each species using the same set of predictors as was used to predict high impact status, but including the number of regions in which a species has been recorded (Table 1). We allowed only a random intercept for each genus. Again we used AICc and AICc weights for model selection.

#### By-sector analysis

We carried out a separate AICc analysis for species that had a high impact within each sector (environmental, pastoral and agricultural). With so few high-impact species for each sector, the traits of each high-impact species could have a disproportionately large effect on the prediction of which species is high-impact (a form of noise fitting). To test against this possibility we carried out a randomisation following the method in the documentation for the lme4 library (see above, and help for ‘simulation’ function in lme4; [41]). We also excluded introduction pathway from the analysis as this categorical predictor had five levels, greatly increasing the number of parameters that had to be estimated, and leading to convergence problems.

## Results

### Overview of Naturalised Grass Flora and their Impacts

We recognise 155 species from five subfamilies as having naturalised in tropical and subtropical Australia (Table S2 and S3). Only 21 species (13.5%) were identified as having a high impact: 13 to the environment, seven to the pastoral industry and five species in agriculture (Table S4). Of these only four (19.0%) were considered high-impact for more than one sector, namely to the environment and pastoral industry (*Eragrostis curvula* and *Hyparrhenia hirta*), and to the environment and agriculture (*Megathyrsus maximus* and *Hymenachne amplexicaulis*).

### Taxonomy and Life History Traits

Naturalised species represent seven grass subfamilies, although all but seven species belong to the Panicoideae (Tribes Paniceae and Andropogoneae) and Chloridoideae (Tribe Cynodonteae) (Table S2). Four of the five poorly represented subfamilies (Arundinoideae, Bambusoideae, Ehrhartoideae and Micrairoideae), together with two panicoid species (*Steinchisma hians* and *Hymenachne amplexicaulis*) are C_3_ species, the remainder being C_4_. Only 10 (6.5%) species are semi-aquatic, the remainder being terrestrial (Table S2). Life histories and growth forms are diverse, even within species (Table S5). Most species were either perennials (60.6%, mostly tufted or rhizomatous) or tufted annuals (28%). Some tufted species also had stolons and/or rhizomes.

### Distribution and Incidence

**Figure 1:**
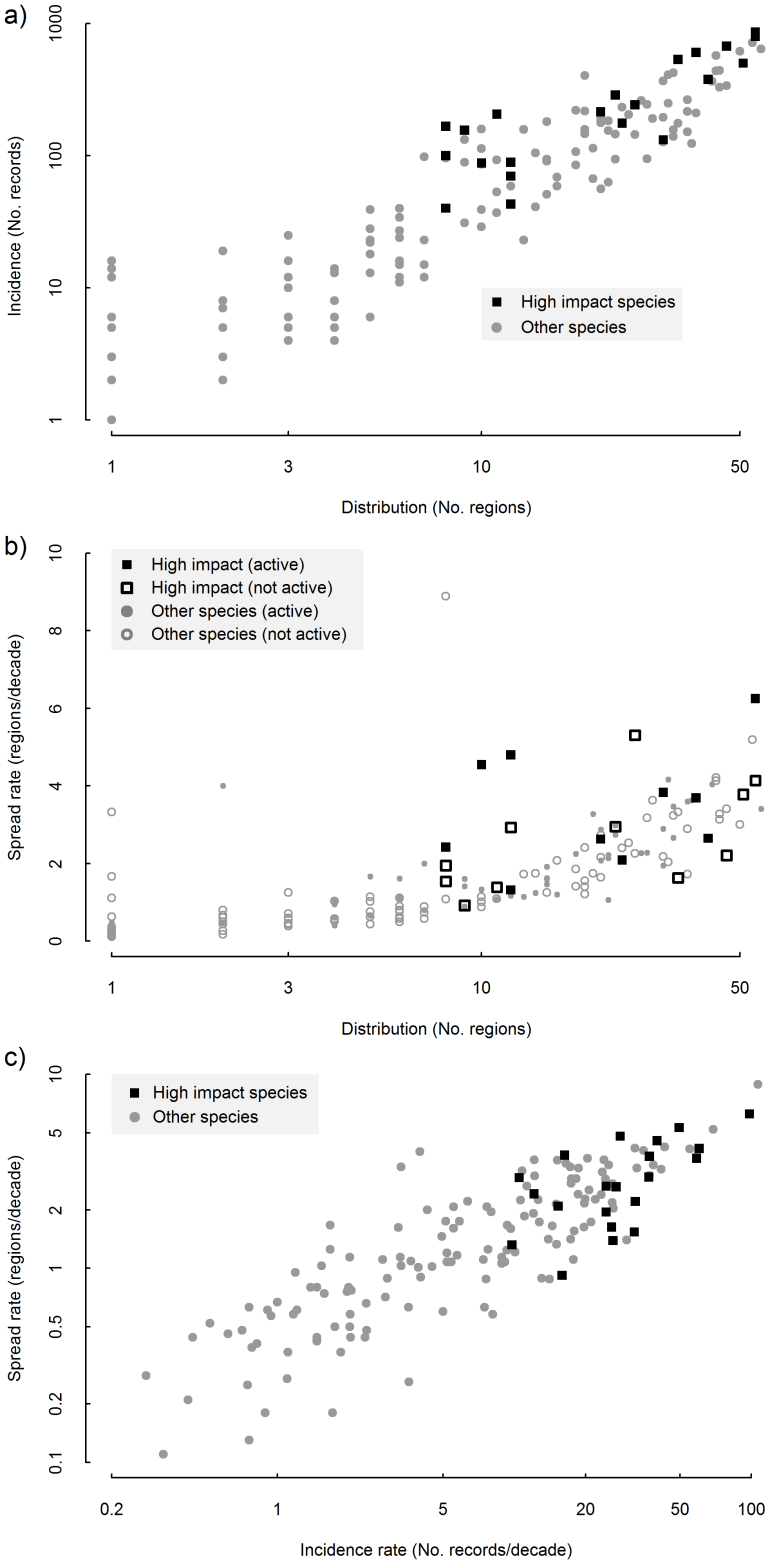
Relationship between distribution and incidence (a) and spread rate (b), and spread rate and incidence rate (c) (n = 155 species). High impact species are shown as squares and actively spread species as closed symbols (b).

Naturalised species on average were recorded from 16 IBRA regions (maximum = 57) and represented by 123 unique herbarium records (maximum = 705). Number of regions was strongly correlated with number of herbarium records (Number of regions = 0.868 x^0.637^, where x = number of records), with no highly-sampled but geographically restricted species (Figure 1a). Spread rate and incidence rate (number of records per decade) were also correlated (Figure 1c). This suggests that distribution, spread rate, incidence (number of records) and incidence rate were all measuring distributional extent, rather than abundance. High-impact species showed the same relationship but with none being localised or poorly sampled (Figure 1a). As a result high-impact species were on average reported from more regions (25.5 vs 14.8) and more often (303 vs 111 records) (Figure 1a).

### Predictors of Impact (All High-impact Species Pooled)

Among naturalised species those having a high impact were more likely to be semi-aquatic, and to have spread more quickly (Table 2). The best model included only these two predictors, they were included among predictors in all top 10 ranked models, and the best model that excluded spread rate performed poorly (Table 2). They were also the best single predictors, although both performed poorly individually (Table 2). The effect of being semi-aquatic can be seen in the raw data: 50.0% of the semi-aquatic species (n = 10) were classified as high-impact, which is much greater than the 13.5% expected if being semi-aquatic had no effect. Likewise, high-impact species included those that had higher spread rates than would be expected from their current distributional extent (Figure 1b). The historical predictors active spread and naturalisation date also appear in many of the top ranked models but added little to model performance, having worse AICc values than the model containing only spread rate and semi-aquatic. Genus had little effect, resulting in a model ranked 367 out of 512 (Table 2). Results were much the same if only species naturalised up to 1988 are included in the analysis (Table S6).

**Table 2:** Best models predicting high-impact species with model performance measured by AICc.

AICc	ΔAICc	AICc weight		Rank	Model
*Fixed effects for the top 10 ranked models*					
47.977	0		0.14	1	spr.rate+semi.aqua
48.704	0.727		0.097	2	spr.rate+semi.aqua+act.spr
49.431	1.454		0.068	3	spr.rate+semi.aqua+nat
49.71	1.733		0.059	4	spr.rate+semi.aqua+act.spr+intro
50.142	2.165		0.047	5	spr.rate+semi.aqua+nat+act.spr
50.637	2.66		0.037	6	spr.rate+semi.aqua+intro
51.281	3.305		0.027	7	spr.rate+semi.aqua+ann.per
51.309	3.333		0.026	8	spr.rate+semi.aqua+nat+act.spr+intro
51.369	3.392		0.026	9	spr.rate+semi.aqua+tuft
51.491	3.515		0.024	10	spr.rate+semi.aqua+rhizo
*Best model without spr.rate*					
56.295	8.318		0.002	60	semi.aqua+act.spr+intro
*Random effect only*					
69.63	21.66		0	367	(1 | genus)
*Top three models with one fixed effect*					
55.262	7.286		0.04	43	spr.rate
61.557	13.581		0	175	semi.aqua
67.027	19.051		0	312	intro

False positives and false negatives are equally weighted in this approach. Model performance was measured by AICc. For all models the random effect is (1|genus). ΔAICc is the difference in AICc between the top ranked model and the model displayed under ‘Model’. AICc weight is a measure of relative support for each model. Rank gives the rank of each model out of the 512 models tested.

Using leave-one-out cross validation we show that the glme’s do have reasonable predictive ability. *Weuc* values for the top ranked models were generally less than 0.5, i.e better than twice as accurate as randomly guessing if a species will be high impact (Table 3). Weighting true and false positives equally, as in the previous analysis, produced much the same result, with spread rate and being semi-aquatic remaining the most important predictors (Table 3). The best models did include additional predictors but this should be viewed with caution as the cross validation test does not explicitly penalise extra predictors in the same way as AICc.

**Table 3:** Best models predicting high-impact species using a statistical learning approach.

*w* = 0.5				*w = *0.9				
*Weuc*	rank		Model	*Weuc*		rank		model
0.383	1		spr.rate+semi.aqua+rhizo	0.480		1		spr.rate+semi.aqua+tuft
0.383	2		spr.rate+semi.aqua+tuft+rhizo+stolon	0.486		2		spr.rate+semi.aqua
0.39	3		spr.rate+semi.aqua	0.486		3		spr.rate+semi.aqua+nat+tuft
0.39	4		spr.rate+semi.aqua+stolon	0.512		4		spr.rate+semi.aqua+nat+stolon
0.39	5		spr.rate+semi.aqua+tuft+rhizo	0.512		5		semi.aqua+act.spr+intro+rhizo
0.39	6		spr.rate+semi.aqua+tuft+stolon	0.513		6		spr.rate+nat+act.spr+intro+tuft+rhizo
0.39	7		spr.rate+semi.aqua+rhizo+stolon	0.519		7		spr.rate+semi.aqua+nat+tuft+rhizo
0.395	8		spr.rate+semi.aqua+tuft+rhizo+nat	0.519		8		spr.rate+semi.aqua+nat+tuft+stolon
0.398	9		spr.rate+semi.aqua+tuft	0.525		9		spr.rate+semi.aqua+nat+rhizo
0.404	10		spr.rate+semi.aqua+intro+tuft	0.525		10		spr.rate+semi.aqua+tuft+rhizo
*Best model without spr.rate*								
0.456	81	semi.aqua+ann.per		0.513	5		semi.aqua+act.spr+intro+rhizo	
*Best model without spr.rate or semi.aqua*								
0.914	267	tuft		0.539	17		act.spr+intro+rhizo	
*Three best single predictor models*								
0.456	80	spr.rate		0.625	80		spr.rate	
0.49	117	semi.aqua		0.787	360		act.spr	
0.548	267	tuft		0.801	383		intro	
*Random effect only*								
0.554	278	(1|genus)		0.898	466		(1|genus)	

Model weighting assumption was tested by comparing true positives and false negatives equally (*w* = 0.5) (comparable to Table 2) and weighting true positives more heavily than false negatives) (*w* = 0.9). *Weuc* is expressed as a proportion of the maximum possible value given the value of *w*, thus in both cases a perfect classifier would have a *Weuc* of 0, and a classifier that is guessing randomly will have a *Weuc* of 1.

When true positives were weighted more strongly than false negatives (*w* = 0.9), to reflect the importance of identifying high impact species, there were some important differences. In general the glmes were poorer classifiers, performing around 50% better than random guessing (right hand *Weuc* in Table 3), as opposed to around 60% better than random guessing when *w* = 0.5 (left hand *Weuc* Table 3). This may be due to the effect of genus, which was included as a random effect in all models. When false positives and true positives were weighted evenly, genus by itself was a reasonable predictor, being nearly twice as good as random guessing (*Weuc = *0.554). However, when true positives were more heavily weighted (*w* = 0.9), genus alone was only marginally better than random guessing (*Weuc* = 0.898). When true positives were weighted higher than false positives, spread rate and semi-aquatic were less dominant. The best model without spread rate was ranked 5^th^ when *w* = 0.9 and 81^st^ when *w* = 0.5 (Table 3). Further, the best model without either spread rate or semi-aquatic was ranked 17^th^ when *w = *0.9 (active spread+intro+rhizo) and 267^nd^ when *w* = 0.5 (tuft).

Using coefficients from the best supported model in Table 2, the probability of being high-impact increased by an average of 0.63 (95% CI: 0.331–1.064) logits for every one region per decade increase in spread rate. This slope is significantly greater than 0. The average probability that a semi-aquatic species was high impact was 0.188 (0.057–0.445); for terrestrial species the average probability of being high impact was 0.029 (0.009–0.054), assuming spread rate was near 0 (i.e. comparing intercepts).

### Predictors of Spread Rate

The best predictors were number of regions and naturalisation date (Table 4). Using coefficients from the best model in Table 4, the relationship between spread rate and year of naturalisation was positive but had a relatively small slope (0.0215). Thus, for every 50 years later a species was naturalised its spread rate increased by 1.07 regions per decade.

**Table 4:** Best models predicting spread rate, model performance measured by AICc.

AICc	ΔAICc	AICc weight	Model
377.296	0	0.494	No.reg+nat
380.035	2.74	0.126	No.reg+nat+semi.aqua
380.219	2.923	0.115	No.reg+nat+act.spr
382.589	5.294	0.035	No.reg+nat+stolon
382.676	5.38	0.034	No.reg+nat+rhizo
382.849	5.553	0.031	No.reg+nat+tuft
383.08	5.784	0.027	No.reg+nat+act.spr+semi.aqua
383.254	5.958	0.025	No.reg+nat+ann.per
384.553	7.257	0.013	No.reg+nat+act.spr+rhizo
385.092	7.797	0.01	No.reg+nat+act.spr+tuft

Model performance was measured by AICc, with log(spr.rate) as the response. For all models the random effect is (1|genus). ΔAICc is the difference in AICc between the top ranked model and the model displayed under ‘Model’. AICc weight is a measure of relative support for each model.

### Predictors of Impact by Sector

Three genera were represented by more than one high-impact species within a sector (Table 5). One of them, *Cenchrus*, was also the best represented among all naturalised species whereas five of the six naturalised *Sporobulus* species were considered to be high-impact. In contrast, naturalised *Paspalum* species were well represented in Australia, but included no high-impact species, and only one out of 15 naturalised *Eragrostis* species (*E. curvula*) was high-impact.

**Table 5:** Comparison of all species and high-impact species by sector.

	All species	High impact species		
		Environmental	Pastoral	Agricultural
Total species	155	13	7	5
Taxonomy				
Most common genera	*Cenchrus (16)*	*Cenchrus* (4)	*Sporobolus* (5)	*Echinochloa* (2)
	*Eragrostis* (14)			
	*Paspalum* (11)			
Traits				
Life history: Peren. & peren./ann.	110 (71.0%)	^#^12 (92.3%)	**^#^7 (100%)**	2 (40.0%)
Habitat: semi-aquatic	10 (6.5%)	^#^ **3 (23%)**	**^#^**0 (0%)	3 (60.0%)
Introduction pathway				
Contaminant	14 (9.0)	1 (7.7%)	5 (71%)	0 (0%)
Invasiveness				
Spread rate (regions/decade)*	1.92±0.11	^#^ **3.49±0.34**	**^#^**1.96±0.35	3.46±0.31
Actively spread	60 (38.7%)	^#^ **10 (76.9%)**	**^#^**2 (29%)	2 (40%)

Only predictors (Table 1) that differed between sectors (see text) are included. Statistical analysis was only possible for environmental and pastoral weeds, and only for a subset of parameters (#). The most influential predictors are indicated in bold. Proportions are given in brackets.

* mean ± SE.

Statistical analyses of predictors of impact within sector were only possible for the environmental and pastoral sector (Table S7). Convergence did not occur for the agricultural analysis as the number of high-impact species was too low (five) and there were no strong patterns.

**Figure 2:**
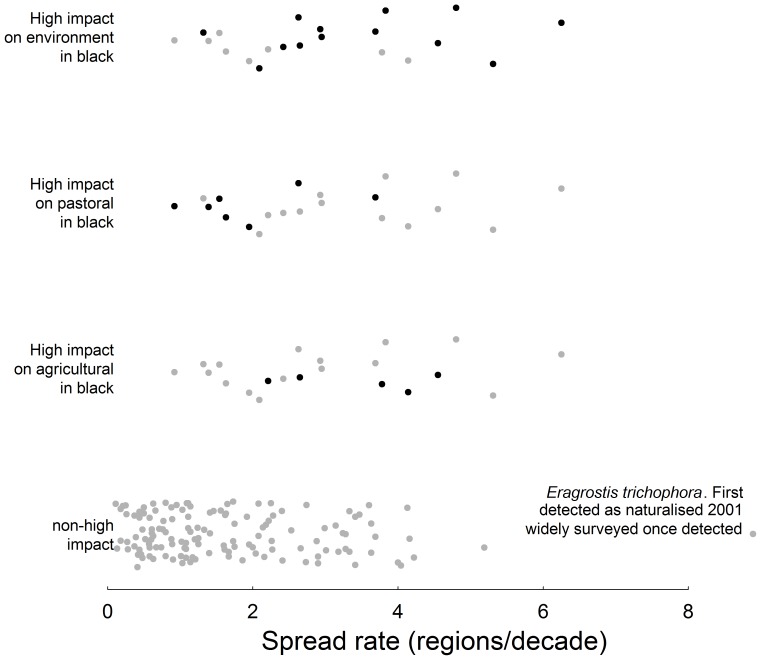
Spread rate of each species (n = 155) including high impact species in each sector. High impact species in each sector are highlighted in separate panels (black dots). Data points are randomly jittered across the y-axis to make visualisation clearer. The very large outlier is explained in the bottom panel.

Among naturalised species, high-impact environmental weeds were more likely to be semi-aquatic (contributing to its importance as a predictor of high-impact species overall, see above), have faster spread rates and be actively spread (Table 5). High-impact environmental species had a wide range of spread rates, including four of the five fastest spreaders (Figure 2), three of which had been actively spread. Being actively spread was by itself an important predictor of high impact, over and above its effect on spread rate (Table S7).

The only significant predictor for high-impact pastoral weeds was life history (Table S7), with all seven species being perennial (Table 5). There were no high-impact pasture species with high spread rates (>4 regions/decade) (Figure 2). Introduction pathway could not be included in the analysis (see methods), but five of the seven (71%) high-impact pastoral species have entered as a contaminant of seeds, compared to only 14% overall. This could, however, be confounded by genus, as all five species were from the same genus, *Sporobolus*.

High-impact agricultural weeds had a high proportion being semi-aquatic, an average spread rate comparable to that of high-impact environmental species, and the lowest proportion of perennial species (Table 5).

## Discussion

At least 1,000 tropical and subtropical grass species are known to have been imported into Australia [27]. Of those, 155 species have naturalised, 115 have spread to at least five biogeographic regions and 21 were identified as high-impact species for the environment or production systems. This is less than a third of the ‘major weeds’ identified in a previous study (n = 64; [21]), in part because the criteria we used required evidence of impact leading to practice change for industry, as well as consideration of the circumstances under which species become dominant in environmental settings. High-impact species were on average no different to all naturalised species in most respects, but had higher spread rates and were more likely to be semi-aquatic. However, spread rates were in turn strongly correlated with other predictors so need to be interpreted cautiously. Although prediction performance was reasonable overall, it declined when attempting to predict high-impact species (minimise false negatives), which is the main focus of biosecurity. Predictive ability is likely to improve greatly if predictions are by sector, but analyses are limited by the relative rarity of high-impact species.

High-impact species are principally the focus of management and policy efforts to limit the impact of invasive plants. However, surprisingly little work has been done to identify them objectively, and mostly this has been restricted to a single sector such as the environment (e.g. [7]). The criteria-based approach we developed allowed us to identify a total of 21 species impacting the environment, pastoral sector or agriculture (cropping and horticulture). Importantly, it explicitly required consideration of the context in which invasion and impact occurs (e.g. historical and current disturbance regimes for environment, and farming practices for production) which is an important determinant of impact [13]. This excluded, for example, many environmental weeds that reach high densities only under human-mediated disturbance regimes. In most cases quantitative data on impacts were lacking, a ubiquitous problem for invasive species [9], [12], [13], and rarely considered context. Nonetheless, our approach did allow short-listing of the 145 species previously recorded as weeds in Australia, and the evidence requirements against each criterion provided a much more rigorous and transparent approach than was previously available. This list will clearly be sensitive to the criteria employed. For example, criteria for high-impact environmental species considered the context, but not the spatial extent (as recommended by [11]), of impact, so species were included that meet the criteria for high environmental impact, but in very restricted settings.

High-impact species were similar to the total naturalised species pool in most respects, although they only comprised species that were widely distributed in Australia (at least eight biogeographic regions), and they were more likely to be semi-aquatic and have higher spread rates when calculated as biogeographic regions per decade. However, spread rate was in turn explained by range size and how recently it had become naturalised in Australia. Range size and spread rate were highly correlated so, as all high-impact species were widely distributed, the correlation between high impact species and spread rate may not be explanatory. Spread rate was also highly correlated with incidence and incidence rate (rate of regions being invaded since naturalisation). Correlations between our measure of spread rate and impact may therefore not be explanatory, which may be why our findings contradict an earlier study which found no correlation between spread rate (measured as km/yr) and impact [7]. Species that became naturalised later spread faster, possibly because spread rates for species that have been naturalised for longer are already approaching their asymptote [46].

Weed risk assessments are used to try to predict what species will become damaging [47]. Our finding that high-impact species have similar characteristics to other naturalised species suggests this task will be difficult. This makes the already difficult problem of correctly identifying relatively rare events (in this case that a naturalised species will become high-impact) [48] much more difficult. Further, risk assessments can be sensitive to how models are optimised in terms of false positives and false negatives, which in turn depends on the application [30]. For example, most analyses weight false positive and false negatives equally, whereas biosecurity is mostly concerned with minimising the risk of missing false negatives (failure to identify a serious threat). Our models were less successful, and required a wider range of predictors, when more weight was given to identifying high-impact species. Previous work has shown that species with congeners considered to be weeds are more likely to have negative impacts [49]. We show that when false negatives are given more weight, genus becomes a very poor predictor, suggesting that using taxonomy as a predictor of impact will be sensitive to how false negatives are weighted. The generality of this result needs to be tested - does it apply to other groups and in other regions? To determine how much weight we should place on detecting true positives (versus avoiding false positives), we need to give careful consideration not only to the risks that exotic species pose, but also the benefits they might bring [29].

Most high-impact species impacted only one sector, none impacted both agricultural and pastoral sectors, and high-impact environmental species included those of great value to the pastoral industry [20], [26], [50]. Furthermore, some species identified as causing high impact to agriculture in a prior study [40] were no longer considered as such due to a change in farming practices (V. Osten, pers. comm.). Similar changes in impact resulting from changes in land management have been observed elsewhere, although most studies focus on changes that increase the threat of invasives [51]. Taken together, these aspects highlight the importance of context in determining impact [9], [13]. As might be expected, different predictors appeared to be important for high-impact species in different sectors. For example, there were differences in life history between sectors, with all pastoral and all but one high-impact environmental species being perennial, compared to only half of high-impact crop-sector species. This is consistent with pasture and environmental weeds needing to out-compete perennial grasses to cause serious impacts in northern Australia [52] (but see [9], who found the annual grass life form to be the best predictor of environmental impact in a global analysis of invasive plants), and annuals being favoured in annual cropping systems. Semi-aquatic species were more likely to be high-impact environmental species, suggesting that semi-aquatic habitats are especially susceptible systems in Australia [24], [26]. Certainly this group included two of the three high-impact species naturalised since 1970, the result of pasture introductions specifically aimed at improving productivity of semi-aquatic pastoral systems [53]. Similar results are apparent for aquatic species [54], although aquatic grass species were not represented in our study. On average, high-impact environmental and agricultural, but not pastoral, species were faster invaders than expected. This could be the result of often relatively well-resourced management programs aimed at containing pasture weeds [17], [55], and the active dispersal of many high-impact environmental species as pasture.

## Conclusions

The importance of avoiding conflation of invasion (spread) with impact [4], [7], and quantifying, explaining, predicting and responding to impact [9], [56], [57] is increasingly being recognised. Our study is one of the first to focus on predictors of species that cause serious impacts and that considers all impacted sectors. Spread rate and habitat were the only universal predictors of impact we found; but even they were not important for each sector. Furthermore, spread rate was difficult to interpret, and does not lend itself to screening tests aimed at identifying a high-impact species, because a plant would have to be widely established before its rate of spread could be measured, and also because it may not be explanatory. Improved predictions will therefore require a deeper understanding of the circumstances in which impact occurs in affected sectors. This represents an important shift of focus for invasion science which to date has focussed largely on predicting invasiveness [5], and on predictors of ecological impacts of invaders [9], [12] rather than on understanding and predicting impacts on environmental or production values, and the circumstances under which those impacts occur. Within the language of risk assessments [8] it suggests greater emphasis is required to characterise consequences of, rather than likelihood of, invasion, as many species are successful invaders yet fail to cause serious impact. Recent calls to shift focus to impacts on ecosystem services (e.g. [13]) represent a shift in the right direction. However, important challenges remain, not least because of the relatively low numbers of high-impact species (low base rates). We expect that the greatest improvements to weed risk assessments will come from developing the theoretical and empirical basis for understanding the circumstances under which some invasive plants cause serious impact to particular sectors.

## Supporting Information

Table S1 **Number of naturalised species reported as weeds in the literature, listed as major weeds by Groves** ***et al*** **. (2003), and that meet our criteria of being high impact species in each of the three sectors we assessed.**
(DOCX)

Table S2 **Subtropical and tropical species that have naturalised in Australia and were included in this study.** Species are grouped by subfamily and tribe, semi-aquatic species are indicated with an asterisk, and high impact species are indicated for the environment (E), pastoral sector (P) and agriculture (A). See text for explanations of each variable. CPI refers to the Commonwealth Plant Introduction List. The complete data set is available from the authors.
(DOCX)

Table S3 **Species that were excluded from this study, and reasons for their exclusion.**
(DOCX)

Table S4 **High-impact species (environmental, pastoral and/or agricultural) and evidence against criteria (see text) required to be classified as such.** Note, in most cases literature on its own wasn’t sufficient to confirm that criteria were met. A wide range of local experts were therefore consulted to determine the nature and circumstances of invasions. We generally only describe one example where criteria are met and do not attempt to synthesise the overall impact in Australia, as this was out of scope.
(DOCX)

Table S5 **Life history and growth form of naturalised species, with high impact species in brackets.** Note that some species are tufted, rhizomatous and/or stoloniferous, or have variable growth forms.
(DOCX)

Table S6 **Best models predicting high-impact species using only species naturalised after 1988, and with model performance measured by AICc.**
(DOCX)

Table S7 **Top 10 models predicting which species have a high impact on the environment or the pastoral sector, ranked by AICc.** For all models* the random effect is (1|genus). ΔAIC is the difference in AICc between the top ranked model and the model displayed under ‘model’. AICc weight is a measure of relative support for each model.
(DOCX)

## References

[1] Mack RN, Simberloff D, Lonsdale WM, Evans H, Clout M, et al (2000) Biotic invasions: Causes, epidemiology, global consequences, and control. Ecological Applications 10: 689–710.

[2] Pimentel D (2002) Biological invasions: economic and environmental costs of alien plant, animal, and microbe species: CRC.

[3] Lowe S, Browne M, Boudjelas S, De Poorter M (2000) 100 of the world’s worst invasive alien species: a selection from the global invasive species database: Invasive Species Specialist Group Auckland, New Zealand.

[4] Richardson DM, Pyšek P, Rejmanek M, Barbour MG, Panetta FD, et al (2001) Naturalization and invasion of alien plants: concepts and definitions. Diversity and distributions 6: 93–107.

[5] Catford JA, Jansson R, Nilsson C (2008) Reducing redundancy in invasion ecology by integrating hypotheses into a single theoretical framework. Diversity and distributions 15: 22–40.

[6] Gurevitch J, Fox G, Wardle G, Taub D (2011) Emergent insights from the synthesis of conceptual frameworks for biological invasions. Ecology Letters 14: 407–418. 21513009 10.1111/j.1461-0248.2011.01594.x

[7] Ricciardi A, Cohen J (2007) The invasiveness of an introduced species does not predict its impact. Biological Invasions 9: 309–315.

[8] Daehler CC, Virtue JG (2010) Likelihood and consequences: reframing the Australian weed risk assessment to reflect a standard model of risk. Plant Protection Quarterly 25: 52–55.

[9] Pyšek P, Jarošík V, Hulme PE, Pergl J, Hejda M, et al.. (2012) A global assessment of invasive plant impacts on resident species, communities and ecosystems: the interaction of impact measures, invading species’ traits and environment. Global Change Biology.

[10] Richardson DM, Van Wilgen BW (2004) Invasive alien plants in South Africa: how well do we understand the ecological impacts?

[11] Parker IM, Simberloff D, Lonsdale W, Goodell K, Wonham M, et al (1999) Impact: toward a framework for understanding the ecological effects of invaders. Biological Invasions 1: 3–19.

[12] Vilà M, Espinar JL, Hejda M, Hulme PE, Jarošík V, et al (2011) Ecological impacts of invasive alien plants: a meta-analysis of their effects on species, communities and ecosystems. Ecology Letters 14: 702–708. 21592274 10.1111/j.1461-0248.2011.01628.x

[13] Hulme PE, Pyšek P, Jarošík V, Pergl J, Schaffner U, et al.. (2012) Bias and error in understanding plant invasion impacts. Trends in ecology & evolution. 10.1016/j.tree.2012.10.010 23153723

[14] Huang QQ, Qian C, Wang Y, Jia X, Dai XF, et al (2010) Determinants of the geographical extent of invasive plants in China: effects of biogeographical origin, life cycle and time since introduction. Biodiversity and conservation 19: 1251–1259.

[15] Huang QQ, Wu JM, Bai YY, Zhou L, Wang GX (2009) Identifying the most noxious invasive plants in China: role of geographical origin, life form and means of introduction. Biodiversity and conservation 18: 305–316.

[16] Butler DW, Fairfax RJ (2003) Buffel grass and fire in a Gidgee and Brigalow woodland: A case study from central Queensland. Ecological Management & Restoration 4: 120–125.

[17] Parsons WT, Cuthbertson EG (2001) Noxious weeds of Australia: Csiro.

[18] Powles SB, Yu Q (2010) Evolution in action: plants resistant to herbicides. Annual Review of Plant Biology 61: 317–347. 10.1146/annurev-arplant-042809-112119 20192743

[19] D’Antonio CM, Vitousek PM (1992) Biological invasions by exotic grasses, the grass/fire cycle, and global change. Annual Review of Ecology and Systematics: Annual Reviews Inc. {a}. pp. 63–87.

[20] Friedel M, Grice A, Marshall N, van Klinken R (2011) Reducing contention amongst organisations dealing with commercially valuable but invasive plants: The case of buffel grass. Environmental Science & Policy.

[21] Groves R, Hosking J, Batianoff G, Cooke D, Cowie I, et al.. (2003) Weed categories for natural and agricultural ecosystem management: Bureau of Rural Sciences Canberra, Australia.

[22] Mack RN (1989) Temperate grasslands vulnerable to plant invasions: characteristics and consequences. Biological invasions: a global perspective: 155–179.

[23] Setterfield SA, Rossiter-Rachor NA, Hutley LB, Douglas MM, Williams RJ (2010) BIODIVERSITY RESEARCH: Turning up the heat: the impacts of Andropogon gayanus (gamba grass) invasion on fire behaviour in northern Australian savannas. Diversity and distributions 16: 854–861.

[24] Ferdinands K, Beggs K, Whitehead P (2005) Biodiversity and invasive grass species: multiple-use or monoculture? Wildlife Research 32: 447–457.

[25] Rossiter NA, Setterfield SA, Douglas MM, Hutley LB (2003) Testing the grass-fire cycle: alien grass invasion in the tropical savannas of northern Australia. Diversity and Distributions 9: 169–176.

[26] Wearne LJ, Clarkson J, Grice AC, Klinken Rv, Vitelli JS (2010) The biology of Australian weeds. 56. Hymenachne amplexicaulis (Rudge) Nees. Plant Protection Quarterly 25: 146–161.

[27] Cook GD, Dias L (2006) TURNER REVIEW No. 12. It was no accident: deliberate plant introductions by Australian government agencies during the 20th century. Australian Journal of Botany 54: 601–625.

[28] Leung B, Roura-Pascual N, Bacher S, Heikkilä J, Brotons L, et al (2012) TEASIng apart alien species risk assessments: a framework for best practices. Ecology Letters 15: 1475–1493. 23020170 10.1111/ele.12003

[29] Yokomizo H, Possingham HP, Hulme PE, Grice AC, Buckley YM (2012) Cost-benefit analysis for intentional plant introductions under uncertainty. Biological Invasions 14: 839–849.

[30] Robinson TP, van Klinken RD, Metternicht G (2010) Comparison of alternative strategies for invasive species distribution modeling. Ecological Modelling 221: 2261–2269.

[31] Mallett K, Orchard A (2002) Flora of Australia Volume 43, Poaceae 1: Introduction and Atlas. ABRS/CSIRO.

[32] Randall RP (2002) A global compendium of weeds: RG and FJ Richardson.

[33] Simon BK, Alfonso Y (2011) AusGrass2. Available: http://ausgrass2.myspecies.info. Accessed 2013 May 10.

[34] Kellogg EA (2002) Synoptic classification of Australian grasses. Flora of Australia 43: 245–248.

[35] Kellogg EA (2009) Synoptic Classification of Australian Grasses. Flora of Australia 44: 1–4.

[36] Simon BK, Clayton WD, Harman KT, Vorontsova MS, Brake I, et al. (2012) Classification of Grasses. Available: http://grassworld.myspecies.info/content/classification-grasses. Accessed 2013 May 18.

[37] Skerman PJ, Riveros F (1990) Tropical grasses: Food & Agriculture Org.

[38] Cook B, Pengelly B, Brown S, Donnelly J, Eagles D, et al.. (2005) Tropical forages: an interactive selection tool. Brisbane: CSIRO/DPI&F (Qld)/CIAT/ILRI 1.

[39] (2007) Australian Plant Herbage Cultivars. Available: http://wwwpicsiroau/ahpc/indexhtm Accessed 2007 January 29.

[40] van Klinken R, Panetta FD, Ross B, Wilson C. A pain in the grass: what’s the diagnosis. 2004: pp. 480–483.

[41] Bates D, Maechler M, Bolker B (2012) lme4: Linear mixed-effects models using S4 classes. R package version 0.999999–0. Available: http://CRAN.R-project.org/package=lme4. Accessed 2013 May 23.

[42] R_Core_Team (2012) R: A language and environment for statistical computing. R Foundation for Statistical Computing, Vienna, Austria. ISBN 3–900051–07–0. Available: http://www.R-project.org. Accessed 2013 May 23.

[43] Warnes MGR (2012) Package ‘gmodels’.

[44] Burnham KP, Anderson DR (2002) Model Selection and Multimodel Inference: A Practical Information-Theoretical Approach. New York, USA: Springer-Verlag.

[45] Bramer M (2007) Principles of data mining. London, UK: Springer-Verlag.

[46] Aikio S, Duncan RP, Hulme PE (2010) Lag-phases in alien plant invasions: separating the facts from the artefacts. Oikos 119: 370–378.

[47] Leung B, Roura-Pascual N, Bacher S, Heikkila J, Brotons L, et al (2012) TEASIng apart alien species risk assessments: a framework for best practices. Ecology Letters 15: 1475–1493. 23020170 10.1111/ele.12003

[48] Caley P, Lonsdale WM, Pheloung PC (2006) Quantifying uncertainty in predictions of invasiveness. Biological Invasions 8: 277–286.

[49] Scott JK, Panetta FD (1993) Predicting the Australian weed status of southern African plants. Journal of Biogeography 20: 87–93.

[50] Lonsdale W (1994) Inviting trouble: introduced pasture species in northern Australia. Australian Journal of Ecology 19: 345–354.

[51] Bradley BA, Blumenthal DM, Wilcove DS, Ziska LH (2010) Predicting plant invasions in an era of global change. Trends in ecology & evolution 25: 310–318. 20097441 10.1016/j.tree.2009.12.003

[52] McIntyre S, Martin T, Heard K, Kinloch J (2006) Plant traits predict impact of invading species: an analysis of herbaceous vegetation in the subtropics. Australian Journal of Botany 53: 757–770.

[53] Wildin J. Aleman grass, Hymenachne and other forage species for ponded pasture systems; 1991.

[54] Forno I, Julien M (2000) Success in biological control of aquatic weeds by arthropods. Biological control: measures of success: Springer. pp. 159–187.

[55] Martin TG, van Klinken RD (2006) Value for money? Investment in weed management in Australian rangelands. The Rangeland Journal 28: 63–75.

[56] Drenovsky RE, Grewell BJ, D’Antonio CM, Funk JL, James JJ, et al (2012) A functional trait perspective on plant invasion. Annals of botany 110: 141–153. 22589328 10.1093/aob/mcs100 PMC3380596

[57] Thomsen MS, Olden JD, Wernberg T, Griffin JN, Silliman BR (2011) A broad framework to organize and compare ecological invasion impacts. Environmental research 111: 899–908. 21696719 10.1016/j.envres.2011.05.024

