# Supplementary material for: Are High-Impact Species Predictable? An Analysis of Naturalised Grasses in Northern Australia
Source: PLoS One. 2013 Jul 9;8(7):e68678. doi: 10.1371/journal.pone.0068678 (PMC3706395; doi:10.1371/journal.pone.0068678)
Supplement: Table S2 — Subtropical and tropical species that have naturalised in Australia and were included in this study. Species are grouped by subfamily and tribe, semi-aquatic species are indicated with an asterisk, and high impact species are indicated for the environment (E), pastoral sector (P) and agriculture (A). See text for explanations of each variable. CPI refers to the Commonwealth Plant Introduction List. The complete data set is available from the authors. (DOCX) [file pone.0068678.s002.docx]

| Species | | First Record (yr) | Naturalisation (yr) | First CPI Record (yr) | Invasion rate | High impact species |
| --- | --- | --- | --- | --- | --- | --- |
| Aristidoideae: Aristideae | |  |  |  |  |  |
|  | *Stipagrostis ciliata* | 1951 | 2004 | 1951 | 1.67 | no |
| Arundinoideae: Arundineae | |  |  |  |  |  |
|  | *Arundo donax* | 1885 | 1908 | NA | 3.63 | no |
| Bambusoideae: Bambuseae | |  |  |  |  |  |
|  | *Phyllostachys aurea* | 1971 | 1971 | NA | 1.03 | no |
|  | *Phyllostachys nigra* | 1885 | 1968 | NA | 0.48 | no |
| Chloridoideae: Cynodonteae | |  |  |  |  |  |
|  | *Chloris gayana* | 1896 | 1906 | 1930 | 4.04 | no |
|  | *Chloris inflata* | 1906 | 1922 | 1931 | 3.18 | no |
|  | *Chloris pilosa* | 1940 | 1942 | 1940 | 0.44 | no |
|  | *Chloris virgata* | 1875 | 1875 | 1929 | 3.78 | A |
|  | *Cynodon aethiopicus* | 1943 | 1944 | 1972 | 0.61 | no |
|  | *Cynodon hirsutus* | 1965 | 1965 | 1972 | 0.44 | no |
|  | *Cynodon incompletus* | 1908 | 1908 | 1931 | 1.08 | no |
|  | *Cynodon nlemfuensis* | 1933 | 1933 | 1972 | 2.99 | no |
|  | *Cynodon radiatus** | 1911 | 1911 | NA | 1.01 | no |
|  | *Cynodon transvaalensis* | 1932 | 1968 | 1932 | 0.95 | no |
|  | *Dactyloctenium aegyptium* | 1868 | 1868 | 1931 | 2.18 | no |
|  | *Dactyloctenium australe* | 1907 | 1907 | 1985 | 1.46 | no |
|  | *Dactyloctenium giganteum* | 1970 | 1971 | 1970 | 1.03 | no |
|  | *Dinebra retroflexa* | 1930 | 1961 | 1930 | 1.02 | no |
|  | *Eleusine indica* | 1844 | 1844 | 1933 | 3.01 | no |
|  | *Eleusine tristachya* | 1882 | 1882 | 1937 | 1.41 | no |
|  | *Eragrostis atrovirens* | 1922 | 1970 | 1957 | 0.50 | no |
|  | *Eragrostis bahiensis* | 1932 | 1960 | 1958 | 0.60 | no |
|  | *Eragrostis barrelieri* | 1883 | 1883 | 1951 | 1.65 | no |
|  | *Eragrostis cilianensis* | 1874 | 1906 | 1934 | 5.19 | no |
|  | *Eragrostis curvula* | 1900 | 1907 | 1931 | 3.69 | EP |
|  | *Eragrostis mexicana* | 1853 | 1853 | 1933 | 1.21 | no |
|  | *Eragrostis minor* | 1903 | 1908 | NA | 3.33 | no |
|  | *Eragrostis paniciformis* | 1971 | 1971 | NA | 0.26 | no |
|  | *Eragrostis pilosa* | 1802 | 1802 | 1930 | 1.73 | no |
|  | *Eragrostis subsecunda* | 1931 | 1931 | NA | 0.13 | no |
|  | *Eragrostis superba* | 1930 | 1933 | 1930 | 0.39 | no |
|  | *Eragrostis tef* | 1909 | 1915 | 1931 | 0.74 | no |
|  | *Eragrostis tenella* | 1888 | 1909 | 1931 | 1.09 | no |
|  | *Eragrostis tenuifolia* | 1930 | 1933 | 1930 | 2.73 | no |
|  | *Eragrostis trichophora* | 2001 | 2001 | NA | 8.89 | no |
|  | *Eustachys distichophylla* | 1897 | 1897 | 1931 | 0.44 | no |
|  | *Leptochloa panicea* | 1931 | 1931 | 1931 | 0.63 | no |
|  | *Perotis indica* | 1980 | 1980 | NA | 0.67 | no |
|  | *Sporobolus africanus* | 1802 | 1802 | 1965 | 1.63 | P |
|  | *Sporobolus coromandelianus* | 1936 | 1936 | NA | 1.08 | no |
|  | *Sporobolus fertilis* | 1912 | 1912 | NA | 0.92 | P |
|  | *Sporobolus jacquemontii* | 1931 | 1931 | NA | 1.39 | P |
|  | *Sporobolus natalensis* | 1911 | 1969 | NA | 1.95 | P |
|  | *Sporobolus pyramidalis* | 1930 | 1958 | 1930 | 1.54 | P |
| Ehrhartoideae: Oryzae | |  |  |  |  |  |
|  | *Oryza sativa** | 1885 | 1956 | 1961 | 1.11 | no |
| Micrairoideae: Isachneae | |  |  |  |  |  |
|  | *Isachne minutula* | 1935 | 1935 | NA | 0.80 | no |
| Panicoideae: Andropogoneae | |  |  |  |  |  |
|  | *Andropogon eucomus* | 1971 | 2007 | 1971 | 3.33 | no |
|  | *Andropogon gayanus* | 1931 | 1985 | 1931 | 4.80 | E |
|  | *Andropogon virginicus* | 1935 | 1935 | 1951 | 1.33 | no |
|  | *Bothriochloa insculpta* | 1934 | 1934 | 1937 | 0.66 | no |
|  | *Bothriochloa pertusa* | 1930 | 1937 | 1930 | 2.88 | no |
|  | *Chrysopogon aciculatus* | 1897 | 1897 | NA | 0.88 | no |
|  | *Chrysopogon zizanioides* | 1930 | 2005 | 1930 | 4.00 | no |
|  | *Coix lacryma-jobi* | 1885 | 1915 | 1930 | 0.42 | no |
|  | *Cymbopogon citratus* | 1965 | 1983 | 1965 | 0.37 | no |
|  | *Cymbopogon martinii* | 1930 | 1954 | 1930 | 0.18 | no |
|  | *Dichanthium annulatum* | 1802 | 1802 | 1931 | 1.06 | no |
|  | *Dichanthium aristatum* | 1912 | 1918 | 1951 | 1.63 | no |
|  | *Dichanthium caricosum* | 1931 | 1937 | 1931 | 0.27 | no |
|  | *Hyparrhenia hirta* | 1930 | 1930 | 1934 | 2.63 | EP |
|  | *Hyparrhenia rufa* | 1930 | 1935 | 1930 | 1.73 | no |
|  | *Mnesithea granularis* | 1890 | 1890 | NA | 1.25 | no |
|  | *Polytrias indica* | 1938 | 1945 | NA | 0.46 | no |
|  | *Saccharum officinarum* | 1788 | 1912 | NA | 0.41 | no |
|  | *Saccharum spontaneum* | 1893 | 1942 | NA | 0.44 | no |
|  | *Schizachyrium microstachyum* | 2001 | 2001 | NA | 1.11 | no |
|  | *Themeda quadrivalvis* | 1932 | 1932 | 1955 | 2.95 | E |
|  | *Tripsacum dactyloides* | 1919 | 1919 | 1940 | 0.11 | no |
|  | *Zea mexicana* | 1929 | 1985 | 1929 | 0.80 | no |
| Panicoideae: Paniceae | |  |  |  |  |  |
|  | *Axonopus compressus* | 1897 | 1897 | 1931 | 1.24 | no |
|  | *Axonopus fissifolius* | 1907 | 1907 | 1948 | 2.14 | no |
|  | *Cenchrus americanus* | 1896 | 1896 | 1931 | 1.14 | no |
|  | *Cenchrus biflorus* | 1930 | 1933 | 1932 | 2.08 | no |
|  | *Cenchrus brownii* | 1910 | 1943 | 1984 | 0.90 | no |
|  | *Cenchrus ciliaris* | 1887 | 1922 | 1930 | 6.25 | E |
|  | *Cenchrus clandestinus* | 1910 | 1910 | 1952 | 3.60 | no |
|  | *Cenchrus echinatus* | 1908 | 1908 | NA | 4.22 | no |
|  | *Cenchrus longisetus* | 1858 | 1887 | 1931 | 2.28 | no |
|  | *Cenchrus longispinus* | 1886 | 1895 | NA | 2.26 | no |
|  | *Cenchrus pedicellatus* | 1940 | 1961 | 1940 | 5.31 | E |
|  | *Cenchrus pennisetiformis* | 1915 | 1915 | 1967 | 3.47 | no |
|  | *Cenchrus polystachios* | 1931 | 1969 | 1931 | 2.93 | E |
|  | *Cenchrus purpureus* | 1916 | 1916 | 1930 | 1.60 | no |
|  | *Cenchrus setaceus* | 1903 | 1903 | 1938 | 2.90 | no |
|  | *Cenchrus setigerus* | 1926 | 1929 | 1938 | 3.83 | E |
|  | *Cenchrus spinifex* | 1913 | 1913 | 1934 | 1.86 | no |
|  | *Cenchrus thunbergii* | 1947 | 1977 | NA | 0.61 | no |
|  | *Digitaria abyssinica* | 1953 | 1983 | 1953 | 0.37 | no |
|  | *Digitaria bicornis* | 1908 | 1908 | NA | 3.24 | no |
|  | *Digitaria ciliaris* | 1843 | 1843 | 1934 | 3.41 | no |
|  | *Digitaria didactyla* | 1903 | 1909 | 1976 | 0.89 | no |
|  | *Digitaria eriantha* | 1930 | 1930 | 1931 | 2.25 | no |
|  | *Digitaria milanjiana* | 1931 | 1973 | 1931 | 1.62 | no |
|  | *Digitaria violascens* | 1888 | 1888 | 1957 | 1.56 | no |
|  | *Echinochloa colona** | 1877 | 1877 | 1931 | 4.14 | A |
|  | *Echinochloa crus-galli** | 1802 | 1802 | 1966 | 2.21 | A |
|  | *Echinochloa crus-pavonis* | 1922 | 1922 | 1970 | 1.14 | no |
|  | *Echinochloa frumentacea* | 1909 | 1909 | 1961 | 2.08 | no |
|  | *Echinochloa oryzoides* | 1931 | 1931 | NA | 0.89 | no |
|  | *Echinochloa polystachya** | 1914 | 1977 | 1950 | 2.42 | E |
|  | *Echinochloa pyramidalis** | 1930 | 1933 | 1930 | 0.52 | no |
|  | *Eriochloa meyeriana* | 1955 | 1968 | 1972 | 0.71 | no |
|  | *Hymenachne amplexicaulis** | 1934 | 1988 | 1934 | 4.55 | EA |
|  | *Megathyrsus maximus* | 1855 | 1855 | 1930 | 2.65 | EA |
|  | *Melinis minutiflora* | 1919 | 1919 | 1930 | 1.32 | E |
|  | *Melinis repens* | 1905 | 1906 | 1930 | 4.13 | no |
|  | *Moorochloa eruciformis* | 1934 | 1956 | 1979 | 1.11 | no |
|  | *Panicum antidotale* | 1911 | 1911 | 1938 | 2.22 | no |
|  | *Panicum coloratum* | 1877 | 1877 | 1936 | 1.20 | no |
|  | *Panicum gilvum* | 1931 | 1931 | 1943 | 2.41 | no |
|  | *Panicum incomtum* | 1868 | 1899 | NA | 0.18 | no |
|  | *Panicum miliaceum* | 1851 | 1851 | 1931 | 1.95 | no |
|  | *Panicum novemnerve* | 1970 | 1970 | NA | 0.25 | no |
|  | *Panicum repens** | 1891 | 1891 | 1943 | 0.50 | no |
|  | *Panicum schinzii* | 1930 | 1930 | 1990 | 1.75 | no |
|  | *Panicum trichoides* | 1874 | 1874 | NA | 1.40 | no |
|  | *Paspalum conjugatum* | 1897 | 1897 | 1931 | 0.88 | no |
|  | *Paspalum dilatatum* | 1876 | 1876 | 1930 | 3.28 | no |
|  | *Paspalum mandiocanum* | 1948 | 1966 | 1948 | 1.14 | no |
|  | *Paspalum nicorae* | 1939 | 1989 | 1947 | 0.48 | no |
|  | *Paspalum notatum* | 1930 | 1932 | 1930 | 1.92 | no |
|  | *Paspalum paniculatum* | 1889 | 1889 | 1948 | 0.58 | no |
|  | *Paspalum plicatulum** | 1914 | 1954 | 1932 | 1.61 | no |
|  | *Paspalum quadrifarium* | 1915 | 1915 | 1960 | 0.63 | no |
|  | *Paspalum urvillei* | 1909 | 1911 | 1946 | 2.53 | no |
|  | *Paspalum vaginatum* | 1801 | 1913 | 1937 | 2.16 | no |
|  | *Paspalum virgatum* | 1917 | 1957 | 1935 | 0.57 | no |
|  | *Setaria barbata* | 1893 | 1962 | NA | 0.21 | no |
|  | *Setaria incrassata* | 1937 | 1975 | 1951 | 2.00 | no |
|  | *Setaria italica* | 1884 | 1886 | 1936 | 2.66 | no |
|  | *Setaria palmifolia* | 1891 | 1907 | 1945 | 1.17 | no |
|  | *Setaria parviflora* | 1903 | 1910 | NA | 2.40 | no |
|  | *Setaria pumila* | 1853 | 1853 | 1963 | 2.04 | no |
|  | *Setaria sphacelata* | 1912 | 1926 | 1929 | 2.74 | no |
|  | *Setaria verticillata* | 1875 | 1875 | 1930 | 3.41 | no |
|  | *Setaria viridis* | 1896 | 1896 | 1945 | 1.75 | no |
|  | *Sorghum arundinaceum* | 1844 | 1908 | 1936 | 1.08 | no |
|  | *Sorghum bicolor* | 1907 | 1907 | 1932 | 3.69 | no |
|  | *Sorghum halepense* | 1870 | 1870 | 1931 | 3.14 | no |
|  | *Sorghum sudanense* | 1920 | 1923 | 1938 | 0.80 | no |
|  | *Sorghum X almum* | 1946 | 1949 | 1946 | 3.28 | no |
|  | *Steinchisma hians* | 1994 | 1994 | NA | 0.63 | no |
|  | *Stenotaphrum secundatum* | 1884 | 1891 | 1930 | 2.27 | no |
|  | *Urochloa brizantha* | 1921 | 1932 | 1929 | 0.77 | no |
|  | *Urochloa decumbens* | 1930 | 1946 | 1930 | 1.41 | no |
|  | *Urochloa fasciculata* | 1914 | 1941 | NA | 0.58 | no |
|  | *Urochloa humidicola* | 1952 | 1980 | 1952 | 1.67 | no |
|  | *Urochloa mosambicensis* | 1933 | 1933 | 1936 | 4.16 | no |
|  | *Urochloa mutica** | 1891 | 1895 | 1963 | 2.09 | E |
|  | *Urochloa oligotricha* | 1972 | 1974 | 1972 | 0.28 | no |
|  | *Urochloa panicoides* | 1930 | 1930 | 1972 | 3.63 | no |
|  | *Urochloa ramosa* | 1986 | 1986 | NA | 1.25 | no |
|  | *Urochloa ruziziensis* | 1944 | 1944 | 1953 | 0.76 | no |
|  | *Urochloa subquadripara* | 1886 | 1886 | NA | 2.90 | no |
|  | *Urochloa texana* | 1907 | 1907 | 1954 | 0.58 | no |
