# Supplementary material for: Are High-Impact Species Predictable? An Analysis of Naturalised Grasses in Northern Australia
Source: PLoS One. 2013 Jul 9;8(7):e68678. doi: 10.1371/journal.pone.0068678 (PMC3706395; doi:10.1371/journal.pone.0068678)
Supplement: Table S4 — High-impact species (environmental, pastoral and/or agricultural) and evidence against criteria (see text) required to be classified as such. Note, in most cases literature on its own wasn’t sufficient to confirm that criteria were met. A wide range of local experts were therefore consulted to determine the nature and circumstances of invasions. We generally only describe one example where criteria are met and do not attempt to synthesise the overall impact in Australia, as this was out of scope. (DOCX) [file pone.0068678.s004.docx]

| **Species** | | **Evidence against criteria** |
| --- | --- | --- |
| Environmental transformer | |  |
|  | *Andropogon gayanus* | Can become dominant in diverse savanna communities under relatively natural disturbance regimes: it does not require soil or canopy disturbance to become established [1] [2]. Listed as one species in the "Threat abatement plan to reduce the impacts on northern Australia's biodiversity by the five listed grasses [3]. |
|  | *Cenchrus ciliaris* | Becomes dominant and forms monocultures across rangeland Australia, including in ungrazed environmental reserves ([4] [5], and references therein). |
|  | *Cenchrus polystachios* | Invades natural bushland where it displaces native plants, resulting in continuous cover beneath an intact canopy[6]. More widespread than *Andropogon gayanus*, and reaches very high density and biomass in environmental reserves including Litchfield National Park [7]. Prevalence on environmental reserves high despite ongoing control efforts [8]. Listed as one species in the "Threat abatement plan to reduce the impacts on northern Australia's biodiversity by the five listed grasses" [3]. |
|  | *Cenchrus pedicellatus* | Behaves similarly to *C. polystachios*. "It has demonstrated its capacity to invade and become prominent in intact native vegetation at numerous sites in the Darwin region. Examples included Cycad study sites (Charles Darwin National Park), Litchfield National Park and open woodland in the Blackmore river area subject to background natural disturbance (occasional fire and native herbivores).” (Dave Liddle, pers. comm., NRETAS Biodiversity Conservation 2012).Listed as one species in the "Threat abatement plan to reduce the impacts on northern Australia's biodiversity by the five listed grasses [3]. |
|  | *Cenchrus setigerus* | Behaves similarly to *C. ciliaris* (A. Mitchell, pers. comm.) |
|  | *Echinochloa polystachya* | Dry tropics (F. Bebawi, pers. comm.) and coastal north Queensland (J. Vitelli, pers. comm.). Potential to form mono-specific stands in seasonally wet and dry areas such as flood plains and swamps where it will smother native species [9]. Invades seasonally flooded areas, swamps and banks of watercourses in Queensland (http://www.daff.qld.gov.au/documents/Biosecurity_EnvironmentalPests/IPA-Aleman-Grass-PP137.pdf). Has become dominant in wetlands above the Barrage (Fitzroy River) under relatively natural (ungrazed) disturbance regimes (J. Clarkson, pers. comm.) |
|  | *Eragrostis curvula* | Becomes dominant in Travelling Stock Reserves (including New England peppermint woodlands) and road reserves, possibly also fits criteria locally in National Parks, especially granite country of the northern Tablelands of New South Wales (W. Whalley pers. comm.; C. Nodallny, pers. comm.). Can become dominant in absence of heavy grazing in very poor country where herbaceous cover is naturally low under trees. |
|  | *Hymenachne amplexicaulis* | Extensively invades native wetlands where it forms monocultures under relatively natural disturbance regimes [10] [9] [11] (and references therein). Listed as one species in the "Threat abatement plan to reduce the impacts on northern Australia's biodiversity by the five listed grasses [3]. |
|  | *Hyparrhenia hirta* | Becomes dominant in woodlands of Kwiambul National Park (on the northwest slopes of New South Wales). This National Park was grazed prior to purchase as an environmental reserve in 1995, but *H. hirta* continues to spread and increase in the park under relatively natural disturbance regimes [12].Becomes dominant in roadside reserves and Travelling Stock Routes and native pastures [12], including where disturbance regimes are relatively natural (W. Whalley, pers. comm.; B. Sindell, pers. comm., C. Nodallny, pers. comm.; Hodginson, pers. comm.). However, we could find no record of dominance in parks/reserves that had not previously been pastoral. In northern Queensland it is becoming dominant under relatively natural disturbance regimes (although on extensive pastoral properties) (J. Clarkson, pers. comm.), although there are questions about its taxonomy there (J. Clarkson, pers. comm., B. Simons, pers. comm.). |
|  | *Megathyrsus maximus* | 42 km of Hervey Bay foreshore invaded, more than half of which is heavily invaded [10], invading dry rainforest [10], invades undisturbed areas in northern New South Wales (D. Officer, pers. comm.), major weed in riparian areas in northern Queensland where it has replaced *Sorghum nitida*, especially in wetter areas (J. Corfield, pers. comm.). Maintains monospecific stands along Mackay’s sandy seashores [13]. However, most commonly associated with high anthropogenic disturbance such as along roadside and forest margins. |
|  | *Melinis minutiflora* | Forms dense monocultures in relatively undisturbed dry sclerophyll forests in southeast Queensland, rapid coloniser of severely burnt sites (such as the foothills around Cairns, northern Queensland). Is driving forest edges back, and forms monocultures in State Forests on the Gillies Ranges under relatively natural disturbance regimes, often in mesic places around granite boulder fields (J. Clarkson, pers. comm.). However, most commonly associated with high anthropogenic disturbance such as along roadside and forest margins. |
|  | *Themeda quadrivalvis* | In Lakefield National Park (northern Queensland) it is replacing and dominating savanna grasslands (including native perennial grasses) under relatively natural disturbance regimes where soil types are favourable (J. Clarkson, pers. comm.). However, gaining dominance most commonly requires bare ground resulting from heavy cattle grazing or poor fire management regimes (J. Vitelli, pers. comm.) |
|  | *Urochloa mutica* | Invades and dominates extensive wetlands in the Northern Territory ([14,15] and references therein). This includes extensive monocultures in the Magela Creek Floodplain on Kakadu National Park which are continuing to expand under relatively natural disturbance regimes [14]. It is "quickly filling in a number of Kakadu's wetlands" (http://www.environment.gov.au/parks/kakadu/management/programs/weeds.html). Listed as one species in the "Threat abatement plan to reduce the impacts on northern Australia's biodiversity by the five listed grasses [3]. |
| Agricultural transformer | |  |
|  | *Chloris virgata* | A major problem in broad-acre, zero-till cropping in central Queensland, where it establishes easily and often grows quickly, requiring high chemical rates for control (M. Conway pers. comm.). It is becoming an increasing problem in zero-till farming as it is not easily controlled by glyphosate (M. Conway, pers. comm.). Also a problem in perennial horticulture in the Sunraysia district of South Australia (V. Osten, pers.comm.). |
|  | *Echinochloa colona* | A serious problem of broadacre cropping in central Queensland, especially in wet areas where it requires high doses of glyphosate to manage (M. Conway, pers. comm.). It is more common than *E. crus-galli* (S. Walker, pers. comm.; V. Osten, pers. comm., and has developed glyphosate resistance (S. Walker, pers. comm.). |
|  | *Echinochloa crus-galli* | A serious problem of broadacre cropping in central Queensland, especially in wet areas where it requires high doses of glyphosate to manage (M. Conway, pers. comm.) |
|  | *Hymenachne amplexicaulis* | Can be a serious problem in sugar cane, where it can grow up and over cane, resulting in contamination and requiring redirection of irrigation channels. This can result in considerable expenses for management, and impacts on farm design (irrigation) [11,16]. |
|  | *Megathyrsus maximus* | A widespread and serious weed of cane field in some areas such as around Maryborough and Bundaberg (Queensland) where it requires specific management (R. Kelly, pers. comm.; T. Linedale, pers. comm.). Its perennial habit and similarity to sugar cane makes it difficult to manage once established. |
| Pastoral transformer | |  |
|  | *Eragrostis curvula* | Without intensive management can reduce the value of pasture ([17], and refs therein). Highly unpalatable (with exception of the Consol variety, or unless carefully managed) and difficult to control. Dominance can result in reducing carrying capacity by at least 3-4 DSE [18]. Weed in black speargrass and *Aristida/Bothriochloa* zones (especially Burnett Region and western Darling Downs) resulting in up to 80% reduction in carrying capacity. Has an ability to invade and dominate native and introduced pastures [19]. Economic loss in Southern Qld, and probably northern Tablelands of NSW (north of Tenterfield). Has invaded and dominated *Phalaris aquatica* pastures south of Braidwood, *Lolium perenne* pastures near Bega, and native grass pastures near Cooma [19]. Many landholders in northern and southern Tablelands and south coast consider it to be a serious weed [19]. Costing New South Wales $6-7 mill/yr [20]. |
|  | *Hyparrhenia hirta* | Becomes dominant under traditional grazing management practices, including in native pastures on the north-west slopes of New South Wales. Has resulted in widespread changes in pastoral practice in affected areas, including through altered grazing management practices [21]. It can be appreciated as a fodder during droughts [21]. |
|  | *Sporobolus africanus* | Dairy industry in Victoria view it as a major problem[18], especially the northern irrigation area where it is invading high input irrigated pasture and severely reducing milk production on heavily infested properties[18]. |
|  | *Sporobolus fertilis* | Pastoral industry considers it causes important losses in northern NSW, although there is considerable variation in what individual people think of it (D. Officer, pers. comm.). Causes serious problems in pastures in the wetter areas on the north coast of New South Wales. It is of low palatability, completely replacing desirable pasture species, with farmers reporting 10-80% losses in carrying capacity. Cattle also take significantly longer to reach equivalent weights compared to those grazing in uninfested pastures (http://keyserver.lucidcentral.org/weeds/data/03030800-0b07-490a-8d04-0605030c0f01/media/Html/Sporobolus_fertilis.htm) |
|  | *Sporobolus jacquemontii* | Recognised as a serious problem in the Burdekin area of northern Queensland, where it is already causing significant reductions in carrying capacity, and changed management practice, although there has been almost no research work done on this species to date (W. Vogler pers. comm.). It can cause serious problems on relatively well-managed properties, although it does best in overgrazed areas (W. Vogler, pers. comm.). |
|  | *Sporobolus natalensis* | Unpalatable, can invade relatively intact pastures, and very difficult to manage once established (W. Vogler, pers. comm.; J. Vitelli pers. comm.). Greatest problems currently in coastal districts of Queensland and northern new South Wales, where it has resulted in substantial reductions in stocking capacity, in cattle taking considerably longer to reach desired weights, high costs in milk production on dairy farms, high management costs, and reduced land values of highly infested lands (http://keyserver.lucidcentral.org/weeds/data/03030800-0b07-490a-8d04-0605030c0f01/media/Html/Sporobolus_fertilis.htm). Anecdotal evidence suggests it can reduce the productivity of beef and dairy enterprises by half, while attempting control can incur major costs [18]. Serious issue in western part of Atherton Tablelands, resulting in changed management practice (W. Vogler pers. comm.). |
|  | *Sporobolus pyramidalis* | Unpalatable, can invade relatively intact pastures, and very difficult to manage once established (W. Vogler, pers. comm.; J. Vitelli pers. comm.). Greatest problems currently in coast Queensland, where it has resulted in substantially reductions in stocking capacity, cattle taking considerably longer to reach desired weights, higher costs in milk production on dairy farms, and reduced land values of highly infested properties (http://keyserver.lucidcentral.org/weeds/data/03030800-0b07-490a-8d04-0605030c0f01/media/Html/Sporobolus_fertilis.htm). Anecdotal evidence suggests it can reduce the productivity of beef and dairy entreprises by half while attempting control can incur major costs [18] |

**Acknowledgements**

Maurice Conway (Qld DPI), Vicki Osten (Qld DPI), Steve Walker (Qld DPI), Rowena Eastik (NT Government), Peter McCosker (Department of Agriculture, WA), various researchers (BSES), Donald Loch (QDPI), Joe Vitelli (QDAFF), Wayne Vogler (QDAFF), David Officer (DPI NSW), John Gavin (NRETAS), Manon Griffith (Qld DPI), Craig Henderson (QDPI), Wright (QDPI), Peter Jeffries (Specialist Weed Control), Greg Lodge (NSW DPI), Wal Whalley (UNE), Chris Nodallny (Office of Environment and Heritage, NSW), Brian Sindel (UNE), Chris Maple (EPA), Ross McCleay, Jeff Corfield (CSIRO), Tony Linedale (BSES), Richard Kelly (BSES), David Francis (Qld), Tim Low (Environmental Consultant), Andrew Mitchell (AQIS), Paul Flower (NSW DEC), Ron Hacker (NSW Agriculture), John Chamberlain (QDPI), Ross Wright (DPI Qld), Ken Hodgkinson (CSIRO), David Liddle (NRETAS), Louise Elliott (NRETAS), Keith Ferdinands (NRETAS), John Hoskings (NSW Agriculture), Dick Pasfield (Ord Land and Water), Bryan Simons (Qld Herbarium) and Faiz Bebawi (QDAFF).

**References**

1. Petty AM, Setterfield SA, Ferdinands KB, Barrow P (2012) Inferring habitat suitability and spread patterns from large‐scale distributions of an exotic invasive pasture grass in north Australia. Journal of Applied Ecology 49: 742-752.

2. Setterfield S, Douglas M, Hutley L, Welch M (2005) Effects of Canopy Cover and Ground Disturbance on Establishment of an Invasive Grass in an Australia Savanna1. Biotropica 37: 25-31.

3. SEWPaC (2011) Draft Threat Abatement Plan to Reduce the Impacts on Northern Australia’s Biodiversity by the Five Listed Grasses. . Canberra.

4. Friedel M, Grice A, Marshall N, van Klinken R (2011) Reducing contention amongst organisations dealing with commercially valuable but invasive plants: The case of buffel grass. Environmental Science & Policy.

5. Grice AC, Friedel MH, Marshall NA, Van Klinken RD (2012) Tackling contentious invasive plant species: A case study of buffel grass in Australia. Environmental management 49: 285-294.

6. Brooks KJ, Setterfield SA, Douglas MM (2010) Exotic grass invasions: applying a conceptual framework to the dynamics of degradation and restoration in Australia’s tropical savannas. Restoration Ecology 18: 188-197.

7. Douglas M, Setterfield S, Rossiter N, Barratt J, Hutley L. Effects of mission grass (Pennisetum polystachion (L) Schult) invasion on fuel loads and nitrogen availability in a northern Australia tropical savanna; 2004. pp. 179-181.

8. Kean L, Price O (2002) The extent of Mission grasses and Gamba Grass in the Darwin region of Australia's Northern Territory. Pacific Conservation Biology 8: 281.

9. Smith NM (2002) Weeds of the wet/dry tropics of Australia-a field guide. Environment Centre NT. Inc, Darwin, Northern Territory, Australia.

10. Low T (1997) Tropical pasture plants as weeds. Tropical Grasslands 31: 337-343.

11. Wearne LJ, Clarkson J, Grice AC, Klinken Rv, Vitelli JS (2010) The biology of Australian weeds. 56. Hymenachne amplexicaulis (Rudge) Nees. Plant Protection Quarterly 25: 146-161.

12. McArdle SL, Nadolny C, Sindel B (2004) Invasion of native vegetation by Coolatai grass Hyparrhenia hirta: impacts on native vegetation and management implications. Pacific Conservation Biology 10: 49.

13. Batianoff GNF, A.J. (1997) Mackay coast: vegetation, floristics and conservation: inventory, management and recommendations. Brisbane.

14. Bayliss P, van Dam R, Bartolo R (2012) Quantitative ecological risk assessment of the Magela Creek Floodplain in Kakadu National Park, Australia: Comparing point source risks from the ranger uranium mine to diffuse landscape-scale risks. Human and Ecological Risk Assessment: An International Journal 18: 115-151.

15. Ferdinands K, Beggs K, Whitehead P (2005) Biodiversity and invasive grass species: multiple-use or monoculture? Wildlife Research 32: 447-457.

16. Csurhes S, Mackey A L. Fitzsimons. 1999. Hymenachne in Queensland. Pest Status Review Series. Queensland Government-Natural Resources and Mines.

17. Firn J, Buckley Y (2007) Understanding the mechanism behind invasion of African lovegrass. TG: Tropical Grasslands 41: 243.

18. Grice AC (2003) Weeds of Significance to the Grazing Industries of Australia: Final Report Prepared for MLA: Meat & Livestock Australia.

19. Campbell M (1983) Area, distribution and weed potential of Eragrostis curvula (Schrad.) Nees in New South Wales. Australian Weeds 2.

20. Hazard W (1988) Introducing crop, pasture and ornamental species into Australia—the risk of introducing new weeds. Australian plant introduction review 19: 19-36.

21. Lodge G, McCormick L, Harden S (2006) Grazing studies of a Hyparrhenia hirta (Coolatai grass) pasture in northern New South Wales. Animal Production Science 45: 1603-1611.
