# Supplementary material for: Are High-Impact Species Predictable? An Analysis of Naturalised Grasses in Northern Australia
Source: PLoS One. 2013 Jul 9;8(7):e68678. doi: 10.1371/journal.pone.0068678 (PMC3706395; doi:10.1371/journal.pone.0068678)
Supplement: Table S6 — Best models predicting high-impact species using only species naturalised after 1988, and with model performance measured by AICc. (DOCX) [file pone.0068678.s006.docx]

| **AICc** | **ΔAICc** | **AICc weight** | | **Rank** | **Model** |
| --- | --- | --- | --- | --- | --- |
| *Fixed effects for the top 10 ranked models* | | | | | |
| 42.948 | 0 | | 0.123 | 1 | spr.rate + semi.aqua |
| 44.001 | 1.053 | | 0.073 | 2 | spr.rate + semi.aqua + ann.per |
| 44.408 | 1.46 | | 0.059 | 3 | spr.rate + semi.aqua + act.spr |
| 44.7 | 1.752 | | 0.051 | 4 | spr.rate + semi.aqua + act.spr + intro |
| 44.81 | 1.862 | | 0.048 | 5 | spr.rate + semi.aqua + ann.per + rhizo |
| 45.07 | 2.122 | | 0.043 | 6 | spr.rate + semi.aqua + nat |
| 45.435 | 2.487 | | 0.035 | 7 | spr.rate + semi.aqua + intro |
| 46.018 | 3.07 | | 0.026 | 8 | spr.rate + semi.aqua + ann.per + intro |
| 46.15 | 3.202 | | 0.025 | 9 | spr.rate + semi.aqua + ann.per + act.spr |
| 46.204 | 3.256 | | 0.024 | 10 | spr.rate + semi.aqua + ann.per + nat |
| *Best model without spr.rate* | | | | | |
| 58.348 | 15.4 | | 0 | 212 | semi.aqua + act.spr + intro |
| *Random effect only* | | | | | |
| 69.64 | 26.69 | | 0 | 338 | (1\|genus) |
| *Top three models with one fixed effect* | | | | | |
| 50.086 | 7.138 | | 0.003 | 52 | spr.rate |
| 63.091 | 20.143 | | 0 | 236 | semi.aqua |
| 68.953 | 26.005 | | 0 | 326 | intro |
