# Supplementary material for: Are High-Impact Species Predictable? An Analysis of Naturalised Grasses in Northern Australia
Source: PLoS One. 2013 Jul 9;8(7):e68678. doi: 10.1371/journal.pone.0068678 (PMC3706395; doi:10.1371/journal.pone.0068678)
Supplement: Table S7 — Top 10 models predicting which species have a high impact on the environment or the pastoral sector, ranked by AICc. For all models* the random effect is (1|genus). ΔAIC is the difference in AICc between the top ranked model and the model displayed under ‘model’. AICc weight is a measure of relative support for each model. (DOCX) [file pone.0068678.s007.docx]

| **AICc** | **ΔAICc** | **AICc weight** | **model** |
| --- | --- | --- | --- |
| *Environmental sector* | | | |
| 25.264 | 0 | 0.189 | spr.rate + semi.aqua + act.spr |
| 27.181 | 1.917 | 0.072 | spr.rate + semi.aqua + act.spr + nat |
| 27.262 | 1.998 | 0.07 | spr.rate + act.spr |
| 27.647 | 2.383 | 0.057 | spr.rate + semi.aqua + act.spr + ann.per |
| 27.696 | 2.432 | 0.056 | spr.rate + semi.aqua + act.spr + stolon |
| 28.433 | 3.169 | 0.039 | spr.rate + semi.aqua + act.spr + tuft |
| 28.439 | 3.175 | 0.039 | spr.rate + semi.aqua + act.spr + rhizo |
| 28.86 | 3.596 | 0.031 | spr.rate + act.spr + nat |
| 29.391 | 4.127 | 0.024 | spr.rate + act.spr + stolon |
| 29.53 | 4.266 | 0.022 | spr.rate + act.spr + ann.per |
| *Pastoral sector* | | | |
| -15.483 | 0 | 0.156 | ann.per + act.spr |
| -14.19 | 1.293 | 0.082 | ann.per + act.spr + stolon |
| -13.705 | 1.777 | 0.064 | ann.per + act.spr + semi.aqua |
| -13.326 | 2.157 | 0.053 | spr.rate + ann.per + act.spr |
| -13.237 | 2.246 | 0.051 | ann.per + act.spr + nat |
| -12.688 | 2.794 | 0.039 | ann.per + act.spr + tuft |
| -12.273 | 3.21 | 0.031 | ann.per + act.spr + stolon + semi.aqua |
| -12.155 | 3.327 | 0.03 | spr.rate + ann.per + act.spr + stolon |
| -12.025 | 3.458 | 0.028 | spr.rate + ann.per + stolon |
| -11.845 | 3.638 | 0.025 | ann.per + act.spr + stolon + nat |

*Method

For each predictor in the best supported model for both sectors (see Table S7) we fitted a reduced model, which was the same as the best model except without the predictor of interest. Then 5000 times we i) we used the ‘simulate’ function in lme4 (Bates *et al*. 2012, lme4: Linear mixed-effects models using S4 classes) to generate a random response vector from the reduced model; ii) using the ‘refit’ function we fitted both the reduced model and the best supported model, with the randomly generated vector as the response; and iii) we recorded the deviance between the reduced model and the best supported model fit to the simulated data. This process generates a distribution of the deviance you would expect to see between the best supported model and the reduced model if the predictor removed from the reduced model was having no effect. The observed deviance between the best supported model and the reduced model was then compared to this null distribution.

Species which had a high impact on the environmental sector were predicted by similar factors as the overall pool of high impact species, with spread rate and semi-aquatic being important predictors (Table S7). The slope for spread rate in the best supported model was 0.77 logits. Semi-aquatic species were 1.95 logits more likely to have a high impact on the environment than terrestrial species. Species that were actively spread were 1.88 logits more likely to be high impact than species that were not actively spread. All these effects are unlikely to be due to chance. Deviance between models with and without spread rate as, large or, larger than the observed was only seen 1 in 900 times in the null distribution. Deviance between models with and without semi-aquatic as large, or larger, than the observed was only seen 1 in 24.5 times in the null distribution. Thus, the effect of being semi-aquatic is probably real, but there is a non-negligible possibility the observed effect is due to chance. Deviance between models with and without actively spread species as large, or larger, than the observed was only seen 1 in 91.5 times in the null distribution.

Species which had a high impact on the pastoral sector were predicted by whether a species was actively spread and if it was annual or perennial. Actively spread species were 11.05 logits more likely to have a high impact on the pastoral sector. However deviance between models with and without actively spread as a predictor as large, or larger, than the observed were seen 1 in 12.7 times in the null distribution. An effect of being actively spread as large as the observed could arise by chance. Perennial species were 20.42 logits more likely to have a high impact on the pastoral sector. Deviance between models with and without life history type as, large, or larger, than the observed was only seen 1 in 34.2 times in the null distribution.
