# Supplementary material for: Are High-Impact Species Predictable? An Analysis of Naturalised Grasses in Northern Australia
Source: PLoS One. 2013 Jul 9;8(7):e68678. doi: 10.1371/journal.pone.0068678 (PMC3706395; doi:10.1371/journal.pone.0068678)
Supplement: Supplementary file 5 [file pone.0068678.s001.docx]

Weed status	Environmental	Pastoral	Agricultural	Total
Weed overseas				153 (98.7%)
Weed in Australia	127	109		145 (93.5%)
Impact				
"Major weed" (Groves *et al*. 2003)	55	37		64 (41.3%)
High impact (our analysis)	13	7	10	25 (16.1%)
Total naturalisations				155
