# Supplementary material for: Are High-Impact Species Predictable? An Analysis of Naturalised Grasses in Northern Australia
Source: PLoS One. 2013 Jul 9;8(7):e68678. doi: 10.1371/journal.pone.0068678 (PMC3706395; doi:10.1371/journal.pone.0068678)
Supplement: Supplementary file 6 [file pone.0068678.s003.docx]

| Species | Criteria for inclusion in the analysis | | | Notes |
|---|---|---|---|---|
| | Subtropical or tropical | Introduced | Naturalised | |
| Alloteropsis cimicina | Yes | Variable | Yes | Considered native, at least in part |
| Alloteropsis semialata | Yes | No | - | Considered native, at least in part |
| Andropogon distachyos | No | Yes | Yes | Temperate based on Australian records |
| Arrhenatherum elatius | No | Yes | Yes | Temperate based on Australian records |
| Bambusa vulgaris | Yes | Yes | ? | Cultivated, couldn't confirm it as naturalised |
| Bothriochloa bladhii | Yes | Variable | Yes | Considered native, at least in part |
| Brachyachne ambigua | Yes | ? | if introduced | Native status unconfirmed. |
| Bromus catharticus | No | Yes | Yes | Primarily temperate although does occur in the tropics |
| Capillipedium spicigerum | Yes | No | - | Native to Australia |
| Cenchrus advena | Yes | Yes | ? | Cultivated, couldn't confirm it as naturalised. |
| Cenchrus caliculatus | Yes | Variable | Yes | Considered native, at least in part |
| Chloris ciliata | Yes | Yes | ? | Couldn't confirm it as naturalised |
| Cortaderia selloana | No | Yes | Yes | Primarily temperate although does occur in the tropics |
| Crypsis schoenoides | No | Yes | Yes | Mediterranean |
| Cynodon dactylon | Yes | Variable | Yes | Considered native, at least in part |
| Digitaria aequiglumis | No | Yes | Yes | Mediterranean or temperate |
| Digitaria insularis | Yes | Yes | ? | Couldn't confirm it as naturalised |
| Digitaria pentzii | - | - | - | Synonym for D. eriantha |
| Digitaria sanguinalis | No | Yes | Yes | Temperate based on Australian records and native range |
| Digitaria ternata | No | Yes | Yes | Temperate based on Australian records and native range |
| Echinochloa esculenta | Yes | No | - | Native |
| Echinochloa microstachya | No | Yes | Yes | Temperate based on Australian records and native range |
| Echinochloa muricata | No | Yes | Yes | Temperate based on Australian records and native range |
| Echinochloa telmatophila | Yes | No | - | Native to Australia |
| Ehrharta erecta | No | Yes | Yes | Temperate based on Australian records |
| Ehrharta longifolia | No | Yes | Yes | Temperate based on Australian records |
| Eleusine coracana | No | Yes | Yes | Temperate based on Australian records |
| Eragrostis infecunda | Yes | Yes | ? | Couldn't confirm it as naturalised |
| Hordeum glaucum | No | Yes | Yes | Temperate based on Australian records |
| Leersia oryzoides | No | Yes | Yes | Native range temperate (but does occur in tropical Australia) |
| Leptochloa fusca | Yes | Variable | Yes | Considered native, at least in part |
| Leptochloa panicea | Yes | Yes | ? | Couldn't confirm it as naturalised |
| Panicum capillare | No | Yes | Yes | Temperate based on Australian and USA records |
| Paspalum distichum | Yes | ? | - | Native status unconfirmed (Groves et al. 2003) |
| Paspalum exaltatum | Yes | Yes | ? | Couldn't confirm it as naturalised |
| Paspalum fasciculatum | Yes | Yes | ? | Couldn't confirm it as naturalised |
| Paspalum scrobiculatum | Yes | Variable | Yes | Considered native, at least in part |
| Pennisetum alopecuroides | Yes | ? | - | Native status unconfirmed (Groves et al. 2003) |
| Pennisetum macrourum | No | Yes | Yes | Temperate based on Australian records and native range |
| Pentaschistis airoides | No | Yes | Yes | Temperate based on Australian records and native range |
| Pentaschistis pallida | No | Yes | Yes | Temperate based on Australian records and native range |
| Pentaschistis thunbergii | No | Yes | Yes | Temperate based on Australian records and native range |
| Phalaris paradoxa | No | Yes | Yes | Temperate based on Australian records and native range |
| Phyllostachys bambusoides | Yes | Yes | ? | Couldn't confirm it as naturalised |
| Puccinellia ciliata | No | Yes | Yes | Temperate based on Australian records and native range |
| Rottboellia cochinchinensis | Yes | Variable | Yes | Probably native to Australia |
| Setaria gracilis | No | Yes | Yes | Temperate |
| Setaria poiretiana | Yes | Yes | ? | Couldn't confirm it as naturalised |
| Sorghum brevicarinatum | Yes | Yes | ? | Couldn't confirm it as naturalised |
| Steinchisma laxa | Yes | Yes | ? | Couldn't confirm it as naturalised |
| Tribolium acutiflorum | No | Yes | Yes | Temperate based on Australian records and native range |
| Tribolium echinatum | No | Yes | Yes | Temperate based on Australian records and native range |
| Tribolium obliterum | No | Yes | Yes | Temperate based on Australian records and native range |
| Tribolium uniolae | No | Yes | Yes | Temperate based on Australian records and native range |
| Uniola paniculata | Yes | Yes | - | Couldn't confirm it as naturalised |
| Urochloa advena | No | Yes | Yes | Temperate based on Australian records |
| Zea mays | Yes | Yes | ? | Couldn't confirm it as naturalised |
| Zoysia matrella | Yes | Variable | Yes | Considered native, at least in part |
