# Supplementary material for: Are High-Impact Species Predictable? An Analysis of Naturalised Grasses in Northern Australia
Source: PLoS One. 2013 Jul 9;8(7):e68678. doi: 10.1371/journal.pone.0068678 (PMC3706395; doi:10.1371/journal.pone.0068678)
Supplement: Supplementary file 7 [file pone.0068678.s005.docx]

Life span	Tufted		Rhizome		Stolon		Total
	yes	variable	yes	variable	yes	variable	
Annual	28% (24%)	0% (0%)	0% (0%)	0% (0%)	1% (0%)	2% (4%)	45 (6)
Annual/Perennial	9% (12%)	1% (0%)	2% (4%)	0% (0%)	1% (0%)	3% (4%)	16 (3)
Perennial	41% (52%)	3% (0%)	28% (24%)	3% (0%)	14% (12%)	8% (4%)	94 (16)
Total	122 (22)	5 (0)	47 (7)	4 (0)	25 (3)	19 (3)	155 (25)
